# Supplementary material for: Causal interplay between lactose intolerance and gut microbiota: a combined bidirectional Mendelian randomization and in vivo validation study
Source: Front Nutr. 2026 Jun 1;13:1803337. doi: 10.3389/fnut.2026.1803337 (PMC13265576; doi:10.3389/fnut.2026.1803337)
Supplement: Supplementary file 1 [file Data_Sheet_1.zip › supplementary materials/Forward/forest plot/ebi-a-GCST90027639.finngen_R12_E4_LACTONAS.pdf]

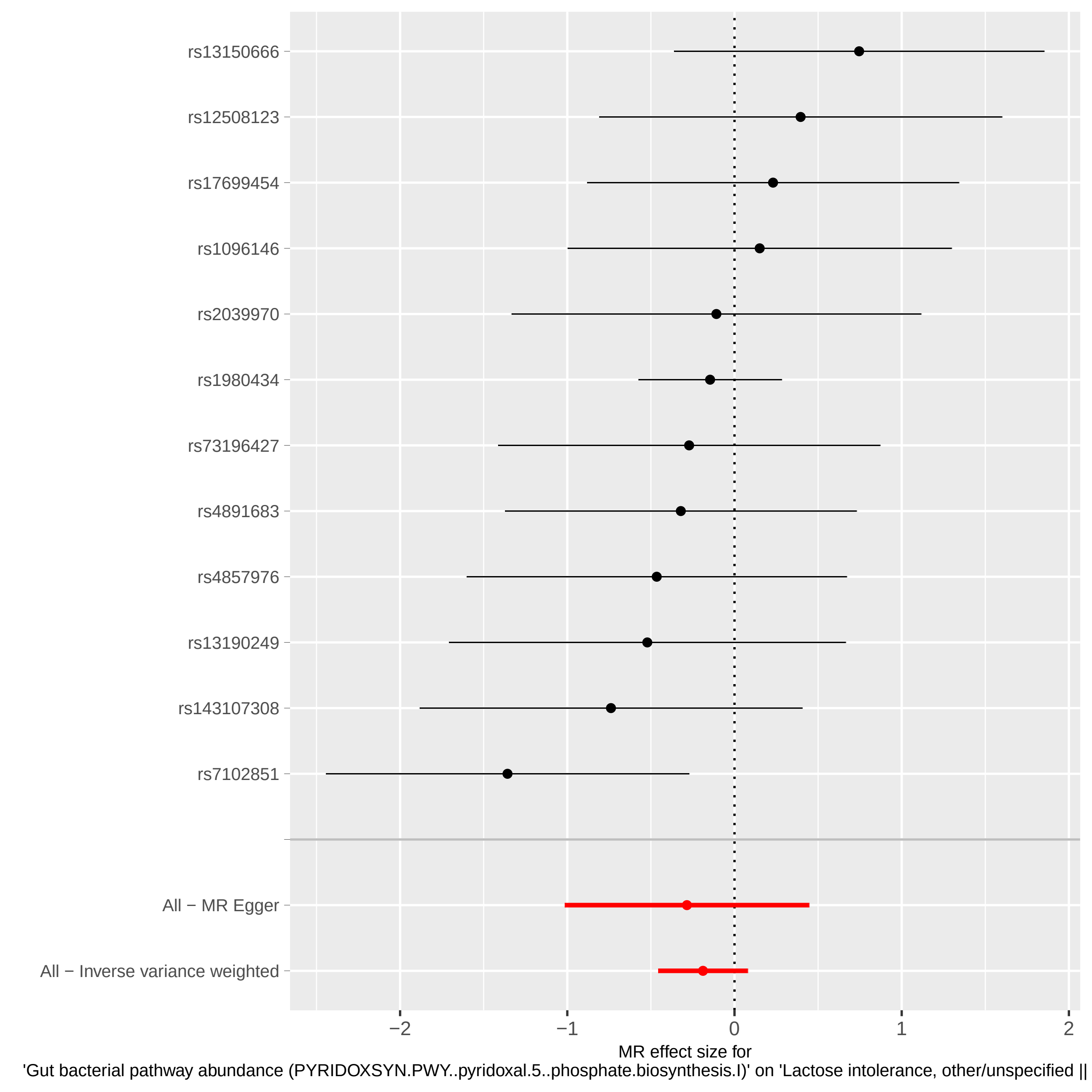

rs13150666
rs12508123
rs17699454
rs1096146
rs2039970
rs1980434
rs73196427
rs4891683
rs4857976
rs13190249
rs143107308
rs7102851
All − MR Egger
All − Inverse variance weighted
−2
−1
0
1
2
MR effect size for
'Gut bacterial pathway abundance (PYRIDOXSYN.PWY..pyridoxal.5..phosphate.biosynthesis.I)' on 'Lactose intolerance, other/unspecified ||
